# The Role of Aminothiols as Biomarkers of Oxidative Stress and Clinical Outcomes in Coronary Artery Bypass Grafting (CABG): A Narrative Review

**DOI:** 10.7759/cureus.96145

**Published:** 2025-11-05

**Authors:** Vladislav Dontsov, Dmitriy A Filimonov, Maxim L Khavandeev, Dmitriy Sobolev, Ivan Karpenko, Grigorii Esion, Abubakar I. Sidik, Dmitriy V Shumakov

**Affiliations:** 1 Department of Cardiothoracic Surgery, M.F. Vladimirsky Moscow Regional Research and Clinical Institute, Moscow, RUS; 2 Department of Experimental Surgery, Gusak Institute of Emergency and Reconstructive Surgery, Donetsk, RUS; 3 Department of Cardiovascular Surgery, Gusak Institute of Emergency and Reconstructive Surgery, Donetsk, RUS; 4 Department of Cardiology, European Medical Center, Moscow, RUS; 5 Department of Cardiothoracic Surgery, A.A. Vishnevsky Hospital, Moscow, RUS; 6 Department of Cardiovascular Surgery, A.A. Vishnevsky Hospital, Moscow, RUS; 7 Department of Cardiovascular Surgery, Peoples' Friendship University of Russia, Moscow, RUS; 8 Department of Cardiovascular Surgery, M.F. Vladimirsky Moscow Regional Research and Clinical Institute, Moscow, RUS

**Keywords:** aminothiols, antioxidant therapies, coronary artery bypass grafting, cysteine, glutathione, homocysteine, oxidative stress, prognosis

## Abstract

This narrative review summarizes current evidence on the role of aminothiols as biomarkers of oxidative stress in patients undergoing coronary artery bypass grafting (CABG). CABG is accompanied by profound oxidative and inflammatory stress driven by ischemia-reperfusion injury, cardiopulmonary bypass (CPB), and systemic immune activation. These processes disrupt redox homeostasis and contribute to myocardial injury, arrhythmogenesis, neurocognitive deficits, and impaired graft patency. Aminothiols, including glutathione, cysteine, homocysteine, and cysteinylglycine, are central regulators of intracellular and extracellular redox balance and have emerged as promising biomarkers in this setting. Evidence from clinical studies indicates that elevated preoperative homocysteine predicts perioperative complications, myocardial infarction, atrial fibrillation, and long-term mortality, while perioperative shifts in glutathione and thiol-disulfide ratios reflect oxidative stress dynamics induced by CPB. Interventional trials suggest that antioxidant therapies such as N-acetylcysteine, melatonin, and vitamin C may favorably modulate aminothiol metabolism, although findings remain inconsistent. Despite small sample sizes, methodological heterogeneity, and lack of standardized cut-offs, aminothiols offer mechanistic and prognostic insights. Their integration into multi-biomarker panels, supported by metabolomics and redox proteomics, could enhance perioperative risk stratification and guide personalized redox-modulating strategies in CABG. By synthesizing mechanistic and clinical data, this review highlights the potential of aminothiols for perioperative risk stratification and redox-modulating interventions.

## Introduction and background

Coronary artery bypass grafting (CABG) is one of the most common cardiac surgical procedures, but it is also associated with substantial oxidative stress and inflammatory burden. During CABG, ischemia followed by reperfusion, cardiopulmonary bypass (CPB), and exposure of blood to artificial surfaces lead to excessive production of reactive oxygen species (ROS) and disruption of antioxidant defenses. These processes can result in endothelial dysfunction, mitochondrial injury, and systemic inflammatory activation that contribute to postoperative complications and adverse outcomes [1,2].

The central pathophysiological mechanism underlying oxidative damage in CABG is ischemia-reperfusion injury. Reintroduction of oxygen into ischemic myocardium leads to a burst of ROS, including superoxide, hydroxyl radicals, and hydrogen peroxide. This oxidative burst triggers mitochondrial permeability transition pore opening, adenosine triphosphate (ATP) depletion, and initiation of apoptosis or necrosis [3]. At the same time, ROS activate redox-sensitive pathways such as nuclear factor kappa-light-chain-enhancer of activated B cells (NF-κB), which amplify inflammation by recruiting neutrophils and other immune cells, further increasing tissue damage. These mechanisms establish a vicious cycle of oxidative stress, redox imbalance, and inflammatory injury, which is a major determinant of myocardial and systemic complications after CABG [4].

Given this burden, there is a need for reliable biomarkers that can reflect the oxidative and inflammatory stress associated with CABG. Conventional biomarkers such as malondialdehyde, 8-isoprostanes, and protein carbonyls have been widely used, but they lack specificity, are influenced by comorbidities, and often fail to capture the dynamic changes of oxidative stress during surgery [5]. This limitation has prompted interest in identifying biomarkers that are mechanistically linked to redox homeostasis and can provide better prognostic information. Aminothiols, such as glutathione (GSH), cysteine, cystine, and homocysteine, are particularly promising because they are directly involved in antioxidant defense and redox regulation [6].

Aminothiols act as both antioxidants and redox sensors. Reduced GSH plays a pivotal role in neutralizing ROS and maintaining cellular redox balance, while the GSH/GSH disulfide (GSSG) ratio is a classic indicator of oxidative stress. Similarly, the cysteine/cystine ratio reflects extracellular redox status. Homocysteine, which is linked metabolically to cysteine via the transsulfuration pathway, has both direct vascular effects and indirect roles in oxidative balance [7]. Alterations in aminothiol levels and their redox ratios are therefore sensitive indicators of oxidative stress burden. Importantly, Patel et al. demonstrated that a composite biomarker of cystine, GSH, and their ratio was independently associated with increased mortality risk in patients with coronary artery disease (CAD), highlighting the clinical relevance of aminothiol redox status [6,7].

Evidence from CABG patients indicates that aminothiol metabolism undergoes marked changes in the perioperative period. Storti et al. observed that serum homocysteine and folate concentrations decreased significantly in the early postoperative period following CABG, suggesting acute disruption of thiol metabolism due to surgical stress [8]. Similarly, a clinical study showed that plasma homocysteine levels dropped to their lowest at about 12 hours after CABG before trending back toward baseline at 48 hours, consistent with transient metabolic shifts [9]. Other reports suggest that higher preoperative homocysteine levels may predict worse long-term graft outcomes, underscoring its potential role as a prognostic biomarker in surgical patients [10].

The appeal of aminothiol biomarkers lies in their mechanistic connection to redox balance and the feasibility of plasma measurement. However, several limitations remain. Analytical challenges such as the need for immediate processing to avoid artifactual oxidation, variability due to comorbidities and nutritional status, and the rapid kinetics of thiol-disulfide changes limit clinical application [11]. Furthermore, robust multicenter studies linking perioperative aminothiol shifts to postoperative outcomes in CABG are still lacking. Therefore, while aminothiols are promising biomarkers of oxidative stress and may have prognostic potential in CABG, further validation in large-scale studies is required before they can be integrated into routine clinical practice [11,12].

This narrative review aims to summarize current evidence on aminothiols as biomarkers of oxidative stress in patients undergoing CABG. We highlight their biological role in redox regulation, perioperative changes during surgery, associations with complications and prognosis, and their potential value in guiding perioperative management and antioxidant therapies.

## Review

Methodology

Relevant articles were identified through targeted searches of PubMed, Scopus, and Google Scholar using combinations of the terms “aminothiol”, “homocysteine”, “glutathione”, “cysteine”, “oxidative stress”, and “coronary artery bypass grafting”. Priority was given to original clinical studies, experimental work relevant to CABG, and recent reviews published between 2000 and 2025. References were selected for their scientific rigor, relevance, and ability to illustrate key mechanisms or clinical associations. This review provides a narrative synthesis of the current state of knowledge.

Oxidative stress in CABG

During CABG, oxidative stress arises through several interconnected mechanisms. Ischemia-reperfusion injury is central to this process: when coronary blood flow is interrupted, the myocardium undergoes anaerobic metabolism with ATP depletion and accumulation of reducing equivalents. Upon reperfusion, the sudden influx of oxygen causes electron leakage in the mitochondrial respiratory chain, leading to the overproduction of ROS such as superoxide, hydroxyl radicals, and hydrogen peroxide. This burst of ROS induces mitochondrial permeability transition pore opening, calcium overload, and activation of apoptotic and necrotic pathways, which contribute to myocardial cell injury [13].

The use of CPB further amplifies oxidative stress. Contact of blood with artificial surfaces, hemolysis, and shear stress release free hemoglobin and catalytic iron that promote radical formation. CPB also activates complement, neutrophils, and endothelial cells, triggering a systemic inflammatory response syndrome (SIRS) that synergizes with ROS to damage vascular and myocardial tissue [14]. The inflammatory activation during CPB sustains oxidative stress through cytokine release, leukocyte recruitment, and endothelial dysfunction, overwhelming endogenous antioxidant systems [13].

The clinical consequences of oxidative stress in CABG are significant. Myocardial injury, often measured by perioperative troponin or creatine kinase-myocardial band (CK-MB) release, correlates with oxidative stress markers [15]. ROS also contribute to the pathogenesis of postoperative atrial fibrillation (POAF) by inducing electrophysiological instability, oxidizing ion channels, and promoting fibrosis and autonomic imbalance [16]. Beyond cardiac complications, oxidative and inflammatory mechanisms have been implicated in neurocognitive decline and delirium after CABG, through microvascular dysfunction, disruption of the blood-brain barrier, and systemic ROS burden [16]. In addition, oxidative modification of lipids and proteins in vascular grafts, together with endothelial dysfunction and intimal hyperplasia, may accelerate atherosclerosis and compromise long-term graft patency [17].

Although endogenous antioxidant defenses, including enzymes such as superoxide dismutase and GSH peroxidase, are activated during CPB, they are frequently insufficient to counteract the oxidative load. Studies have shown that antioxidant enzyme activity may rise transiently, yet the overall redox balance remains shifted toward oxidative damage due to consumption of thiols and accumulation of oxidized products [16,18]. This imbalance underlines the role of oxidative stress as a key driver of myocardial, neurological, and vascular complications in patients undergoing CABG.

Aminothiols and redox biology

A range of studies have examined the role of aminothiols in patients undergoing CABG, evaluating their prognostic significance, perioperative dynamics, and response to therapeutic interventions. Table 1 summarizes the principal findings, including the analytes measured, sample types, and their associations with clinical outcomes.

**Table 1:** Summary of Clinical Studies Evaluating Aminothiols as Biomarkers of Oxidative Stress and Outcomes in Patients Undergoing CABG. bGSH: reduced glutathione in blood; GSSG: glutathione disulfide (oxidized glutathione); RS GSH: redox status of glutathione (2·bGSH/GSSG); tGSH: total glutathione; tCys: total cysteine; tHcy: total homocysteine; tCG: total cysteinylglycine; SAM: S-adenosylmethionine; SAH: S-adenosylhomocysteine; Hb: hemoglobin; CAD: coronary artery disease; CABG: coronary artery bypass grafting; CPB: cardiopulmonary bypass; AF: atrial fibrillation; MI: myocardial infarction.

Study	Analyte	Sample type	Findings	Clinical associations
Ivanov et al., 2023 [2]	Glutathione (bGSH, GSSG, RS GSH, tGSH); cysteine; homocysteine; cysteinylglycine	Blood (plasma, whole blood)	CABG disrupts thiol correlations; postoperative loss of preoperative associations; increased oxidative stress	Associations with perioperative glucose and redox imbalance
Shafiei et al., 2018 [19]	Indirect (via NAC effect on glutathione availability)	Plasma biomarkers (MDA, lactate, troponin I, TNF-α)	NAC and melatonin reduced oxidative stress markers perioperatively	Shorter ICU and hospital stay; improved oxidative stress profile
Shammas et al., 2008 [20]	Homocysteine	Plasma	Elevated preoperative homocysteine predicted cardiovascular death, nonfatal MI, and graft disease at 2 years	Independent prognostic marker of adverse outcomes
Panicker et al., 2023 [21]	Homocysteine	Plasma	Hyperhomocysteinemia associated with acute postoperative complications	Utility in perioperative risk stratification
Girelli et al., 2006 [22]	Homocysteine	Plasma	Fasting homocysteine ≥25.2 µmol/L strongly predictive of cardiovascular and all-cause mortality at 5 years	Independent determinant of long-term prognosis
Cziraki et al., 2023 [23]	Native thiols, total thiols, disulfides, thiol/disulfide ratios	Plasma	Perioperative thiol–disulfide homeostasis shifts with CPB, showing reduced thiols and increased disulfides	Reflects oxidative stress burden; potential biomarker of redox imbalance
Hill et al., 2019 [24]	Indirect (thiol-disulfide balance via vitamin C)	Plasma oxidative stress markers	Vitamin C reduced oxidative stress markers during CABG and shifted thiol/disulfide balance	Demonstrates utility of redox monitoring for antioxidant therapy
Zakkar et al., 2015 [14]	Oxidative stress-related thiol markers	Blood/experimental models	Oxidative stress and inflammation identified as central drivers of postoperative AF	Thiol markers could stratify risk of AF and graft dysfunction
Sun et al., 2021 [25]	Homocysteine effects on KCa channels (BKCa, IKCa, SKCa)	Internal mammary artery (ex vivo)	Homocysteine suppressed BKCa-mediated vasodilation by downregulating the β1-subunit; enhanced TXA2-mediated constriction; compensatory activation of IKCa/SKCa channels	Potential risk of impaired graft flow and vasospasm; relevance to graft patency in CABG patients with hyperhomocysteinemia
Xiong et al., 2025 [26]	Homocysteine	Plasma	Elevated preoperative homocysteine was independently associated with MACE after CABG; Kaplan–Meier analysis confirmed higher risk across unadjusted, PSM, and IPTW models	Strong prognostic biomarker for adverse outcomes; potential role in perioperative risk assessment

Aminothiols, which include cysteine, homocysteine, cysteinylglycine, and GSH, are small sulfur-containing molecules that play an essential role in maintaining cellular redox homeostasis. Cysteine is the most abundant low-molecular-weight thiol in plasma and exists primarily in oxidized or protein-bound forms. Homocysteine, a byproduct of methionine metabolism, is closely linked to cysteine through the transsulfuration pathway and can exert pro-oxidant effects when accumulated. Cysteinylglycine is derived from GSH degradation, while GSH itself, a tripeptide composed of glutamate, cysteine, and glycine, is considered the most important intracellular antioxidant [27].

According to Georgiou-Siafis and Tsiftsoglou [28], the GSH system is central to detoxification and redox balance, with reduced GSH neutralizing ROS either directly or through enzymatic activity. When oxidized, GSH forms GSSG, which is subsequently reduced back to GSH by glutathione reductase using nicotinamide adenine dinucleotide phosphate (NADPH). This cycle not only preserves intracellular reducing capacity but also prevents irreversible oxidation of proteins by allowing thiol-disulfide exchange reactions. A study by Aquilano et al. [29] further emphasized that GSH is more than a passive antioxidant; it also serves as a redox signal mediator by modulating protein S-glutathionylation, thereby influencing gene expression and enzyme activity.

Redox couples such as the GSH/GSSG and Cys/CySS ratios provide sensitive indicators of oxidative stress. According to McBean et al., a decline in the GSH/GSSG ratio reflects oxidative overload at the intracellular level, whereas the Cys/CySS couple mirrors extracellular or plasma redox status [30]. Similarly, Ueland et al. proposed that the collective measurement of reduced, oxidized, and protein-bound aminothiols constitutes a “redox thiol status,” representing an important element of extracellular antioxidant defense [27].

Clinical and experimental studies confirm the sensitivity of aminothiol pools to oxidative disturbances. For instance, a study by Ivanov et al. demonstrated that reduced aminothiols such as cysteine, homocysteine, and GSH significantly decreased during acute ischemia, with partial recovery in reperfusion phases [31]. According to Maksimova et al., disturbances in aminothiol/disulfide homeostasis have also been observed in patients with cardiovascular and renal disease, highlighting that alterations in thiol pools are not merely biochemical findings but also clinically relevant indicators of redox imbalance [32].

Evidence of aminothiols in patients undergoing CABG

Preoperative aminothiol status appears to mirror baseline cardiovascular risk and redox vulnerability in patients awaiting CABG. For instance, a study by Ivanov et al. found that patients with CAD scheduled for surgery had lower intracellular blood GSH and an altered redox status compared with healthy controls, with negative correlations between homocysteine or cysteine and intracellular GSH at baseline, suggesting depletion of the intracellular antioxidant pool in the presence of higher circulating thiols [2]. According to Panicker et al., preoperative hyperhomocysteinemia predicted immediate postoperative adverse events after CABG, supporting the view that elevated homocysteine identifies a high-risk phenotype even before the operative insult [21]. Longer-term data also indicate prognostic relevance.

A study by Shammas et al. reported that elevated homocysteine on admission predicted cardiovascular death, nonfatal myocardial infarction, and symptomatic graft disease at two years following CABG [20]. In a cohort of 350 individuals followed for nearly five years, markedly elevated fasting homocysteine (≥25.2 µmol/L, above the 90th percentile) was associated with significantly higher cardiovascular and all-cause mortality compared to patients with lower levels. This association remained robust after adjustment for renal function, vitamin status, and the MTHFR 677C→T polymorphism, suggesting that extreme elevations of homocysteine independently predict adverse outcomes. Moderate increases in homocysteine (15-25 µmol/L), however, did not carry the same prognostic weight, indicating that only high-level hyperhomocysteinemia is strongly predictive of poor outcomes [22].

Intraoperative and early perioperative changes in aminothiols have been demonstrated, with CPB acting as a key modifier. According to Zakkar et al., CPB activates inflammatory and coagulation pathways and shifts the redox state, which provides a mechanistic basis for thiol perturbations during surgery [33]. A study by Storti et al. showed that homocysteine and folate concentrations fell significantly in the early postoperative period after CABG, consistent with acute consumption or redistribution of these metabolites under oxidative stress conditions [8]. Similarly, a study by Farouk et al. found that homocysteine fell significantly after CABG, while several oxidative stress surrogates changed in a direction consistent with an acute oxidative and inflammatory response [34]. In contrast, a study by Jeremy et al. observed that homocysteine increased at six days and six weeks after conventional on-pump CABG, which indicates that early reductions may be followed by subacute rises as recovery progresses and micronutrient handling changes, including copper and ceruloplasmin dynamics [35]. Taken together, these reports point to time-dependent and context-dependent effects of CPB and reperfusion on plasma aminothiols. Associations between aminothiol levels and oxidative stress markers in CABG patients are summarized in Table 2 [2], which highlights correlations between blood GSH and circulating thiols.

**Table 2:** Pearson correlations between aminothiol levels in controls, CAD patients before and after CABG The table highlights CAD-specific negative associations (e.g., bGSH with tHcy and tCys) that disappear post CABG, indicating disrupted aminothiol metabolism. * Pearson correlation coefficient. bGSH: reduced glutathione in blood (intracellular pool, ~99% of GSH is in this form); GSSG: oxidized glutathione (disulfide form); bGSH/GSSG (a recognized index of oxidative stress); tCys: total cysteine (plasma cysteine including reduced and oxidized forms); tHcy: total homocysteine (reduced + oxidized); tCG: total cysteinylglycine (a dipeptide formed during GSH metabolism); SAM: S-adenosylmethionine (a methyl donor in the methionine cycle); SAH: S-adenosylhomocysteine (precursor of homocysteine); Hb: hemoglobin (used for normalization, e.g., bGSH/Hb ratio); CAD: coronary artery disease; CABG: coronary artery bypass grafting; NS: not significant (statistical result). Table Credit: Ivanov et al., 2023 [2]; published under CC BY 4.0, Attribution 4.0 International Deed (https://creativecommons.org/licenses/by/4.0/)

Indicators	Controls R*	Controls p	CAD R*	CAD p	CABG R*	CABG p
bGSH and GSSG	0.607	0.002	0.354	NS	0.773	<0.001
bGSH and tCys	−0.538	0.011	−0.429	0.043	0.097	NS
bGSH/Hb and tCys	-	-	−0.415	0.045	0.091	NS
bGSH and tHcy	0.217	NS	−0.545	0.018	0.006	NS
bGSH/Hb and tHcy	-	-	−0.531	0.025	0.093	NS
bGSH and SAH	−0.563	0.005	−0.078	NS	0.225	NS
GSSG and tCys	−0.551	0.011	−0.275	NS	0.213	NS
tCys and tCG	0.372	NS	0.505	0.043	0.354	>0.05
tCys and tHcy	0.214	NS	0.743	<0.001	0.354	NS
tCys and SAM	0.049	NS	0.707	<0.001	−0.045	NS
tCys and SAH	0.199	NS	0.565	0.018	0.117	NS
tCys and tGSH	−0.484	0.028	0.150	NS	0.067	NS
tHcy and SAM	−0.27	NS	0.546	0.019	0.375	NS
tHcy and SAH	−0.078	NS	0.664	0.001	0.462	NS
tGSH and SAM	−0.181	NS	0.218	NS	−0.522	0.042

**Figure 1:**
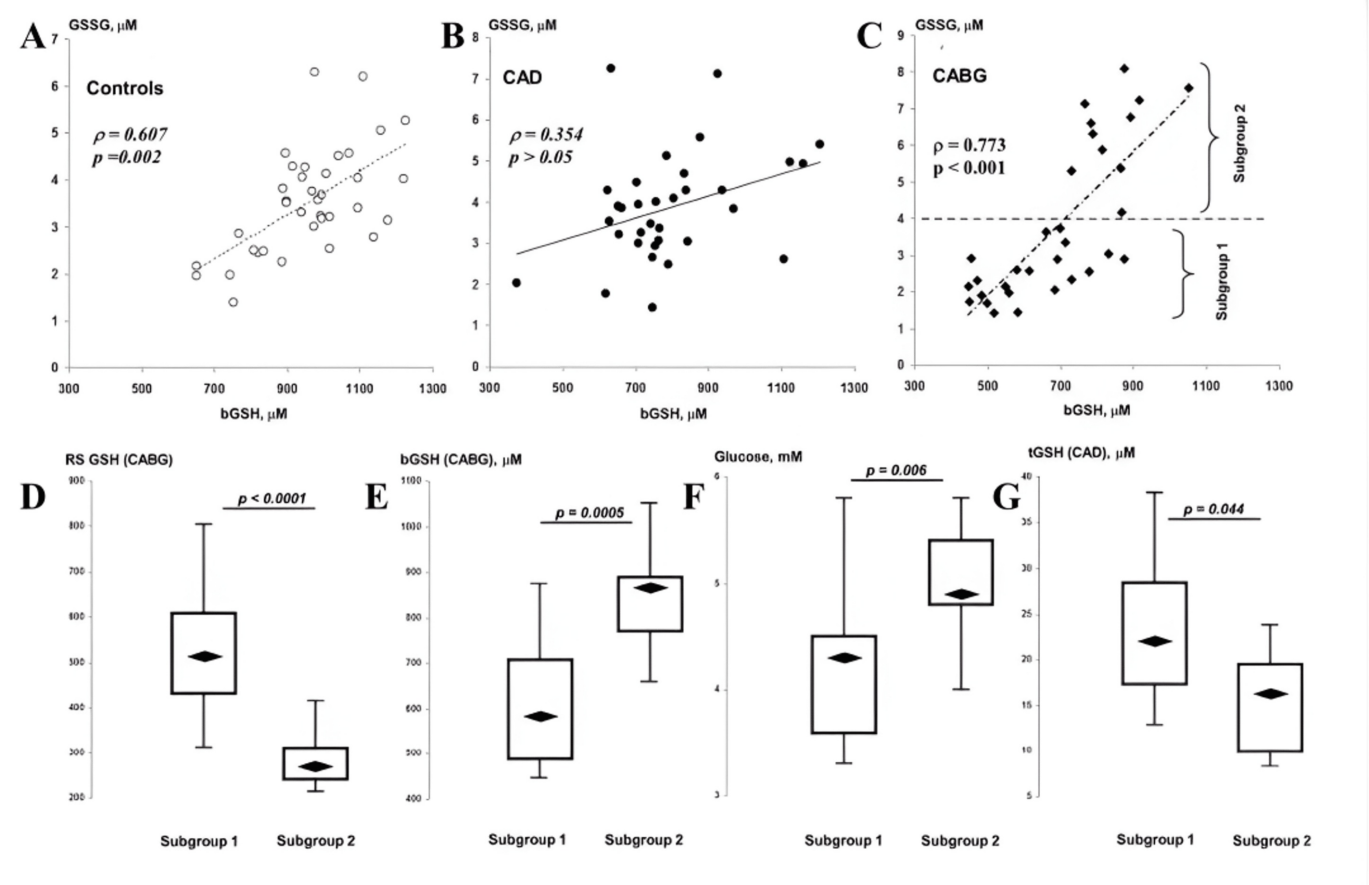
Postoperative dynamics of aminothiols Associations of bGSH with GSSG in controls (A), CAD patients pre-CABG (B), and post-CABG (C). Distributions of postoperative RS GSH (D) and bGSH (E), preoperative glucose (F), and tGSH (G) in post-CABG subgroups divided by GSSG levels (subgroup 1: low GSSG. bGSH: reduced glutathione in blood (intracellular pool, ~99% of GSH is in this form); GSSG: oxidized glutathione (disulfide form); RS GSH: redox status of glutathione, calculated as 2·bGSH/GSSG (a recognized index of oxidative stress); tGSH: total glutathione in plasma (includes both reduced and oxidized forms); CAD: coronary artery disease; CABG: coronary artery bypass grafting; NS Image Source: Ivanov et al., 2023 [2]; published under CC BY 4.0, Attribution 4.0 International Deed (https://creativecommons.org/licenses/by/4.0/)

Postoperative dynamics of aminothiols reveal disruption of preoperative coupling between extracellular thiols and intracellular GSH and show correlations with metabolic and oxidative stress markers. For instance, the study by Ivanov et al. reported that the preoperative negative associations between homocysteine or cysteine and intracellular GSH disappeared after surgery, while increases in GSSG correlated with fasting glucose, implying that perioperative glucose control may modulate GSH oxidation in CABG patients [2]. As illustrated in Figure 1, the negative associations between homocysteine, cysteine, and blood GSH present preoperatively were lost after CABG, and postoperative increases in GSSG correlated with fasting glucose [2]. According to Zakkar et al., the inflammatory response of CPB and reperfusion provides the biological context for such shifts, since leukocyte activation and endothelial dysfunction are tightly linked with thiol oxidation and disulfide formation [33].

Dynamic perioperative changes in thiol-disulfide homeostasis have been confirmed in prospective cohorts. In one study, plasma levels of native thiols and total thiols declined significantly during CPB, accompanied by increases in disulfides and shifts in thiol-disulfide ratios, reflecting acute oxidative stress [32]. These trends persisted into the early postoperative period, suggesting incomplete restoration of redox equilibrium.

**Figure 2:**
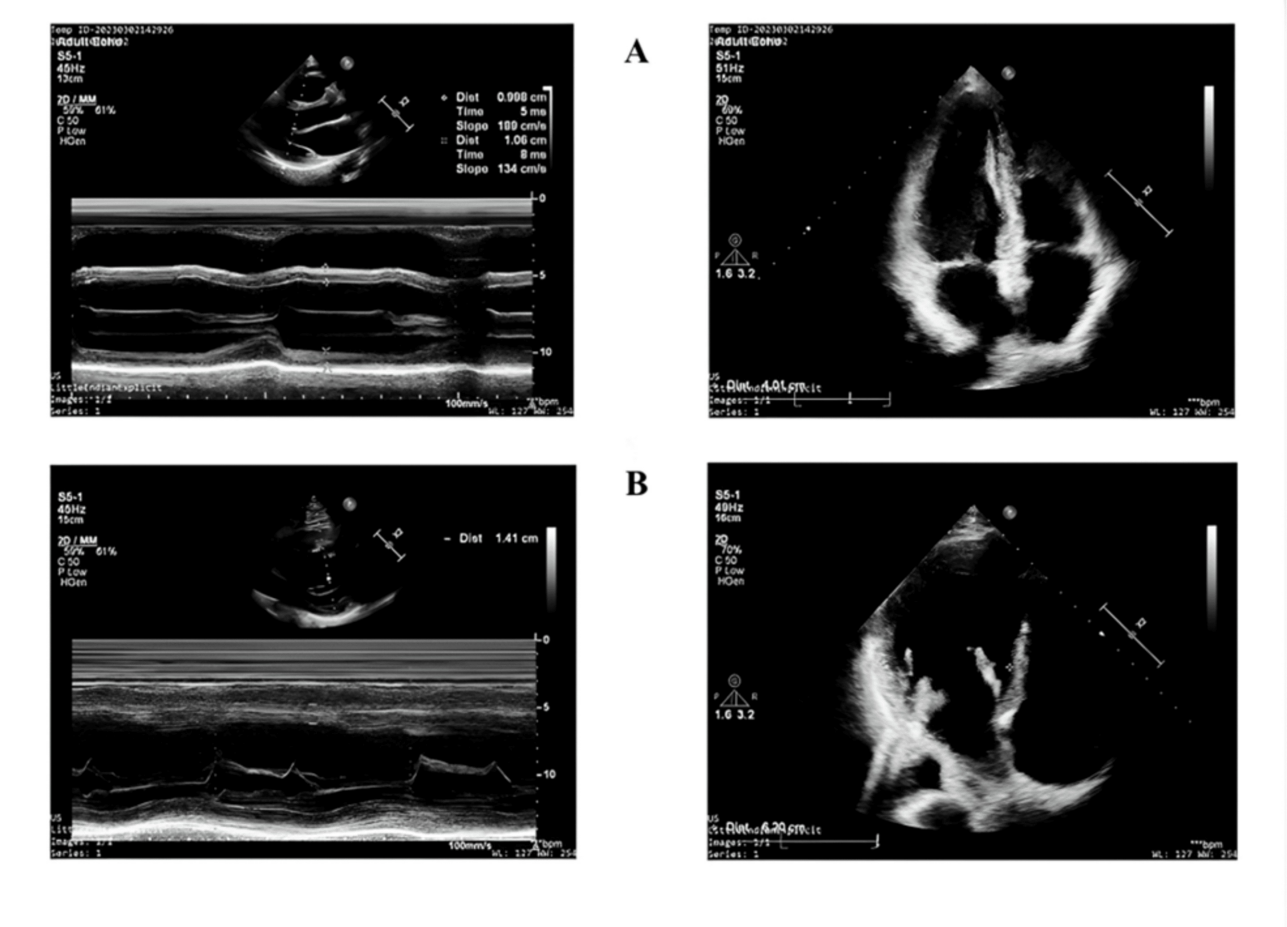
Hypertrophic changes associated with aminothiol dysregulation (A) Two-dimensional transthoracic echocardiographic image of a CABG patient with tHcy < 12 µM/L. Left panel: parasternal long axis view: normal thickness of IVS and posterior wall can be seen (10 mm). Right panel: Apical four-chamber view shows a normal LVED (40 mm). (B) Two-dimensional transthoracic echocardiographic image of a CABG patient with tHcy > 12 µM/L. Left panel: Parasternal long axis view: considerably increased (14 mm) thickness of IVS and posterior wall can be seen. Right panel: Apical four-chamber view shows an increased LVED (60 mm). LVED: left-ventricular end-diastolic diameter; CABG: coronary artery bypass grafting; tHcy: total homocysteine (reduced + oxidized); IVS: interventricular septum Image Credit: Cziraki et al., 2023 [23]; published under CC BY 4.0, Attribution 4.0 International Deed (https://creativecommons.org/licenses/by/4.0/)

Aminothiol dysregulation extends to systemic complications. Low cysteine and γ-glutamylcysteine (γ-GC) availability, often due to impaired transsulfuration or reduced cystine uptake via the xCT transporter, limits GSH synthesis, promoting acute kidney injury (AKI) (incidence 5-30%) through oxidative tubular damage [36,37]. Elevated cystine, the oxidized form of cysteine, reflects a pro-oxidant state and is associated with prolonged ICU stays [38]. In the long term, a persistently high cystine/GSH ratio or low γ-GC levels, observed in cohorts followed for two to five years, predict recurrent cardiovascular events (hazard ration (HR) 1.92) and mortality by sustaining a pro-oxidant environment that drives atherosclerosis progression [39]. This remodeling is visually evident in echocardiographic assessments, where elevated total homocysteine (tHcy) is associated with hypertrophic changes (Figure 2), suggesting a mechanistic link between homocysteine-driven oxidative stress and structural cardiac alterations. In CABG, on-pump procedures amplify these effects compared to off-pump techniques, as CPB-induced inflammation and ROS burst further deplete GSH, γ-GC, and cysteinylglycine while elevating oxidized aminothiols like cystine and GSSG, linking mechanistic dysregulation to clinical outcomes like POAF, AKI, and graft failure [23,37].

Postoperative alterations in aminothiol metabolism have been linked with myocardial injury and redox imbalance. Homocysteine levels were shown to rise significantly after CABG, typically within one to six weeks following surgery, independent of renal function or folate status. Importantly, these elevations correlated closely with markers of myocardial injury such as troponin T, suggesting that homocysteine may serve not only as a byproduct of disturbed redox metabolism but also as a sensitive indicator of perioperative myocardial stress. Furthermore, a direct comparison of plasma and pericardial fluid samples revealed a strong correlation in homocysteine levels, while troponin-I concentrations were nearly tenfold higher in the pericardial compartment, highlighting its role as a more sensitive reservoir for detecting subclinical myocardial injury [26].

**Figure 3:**
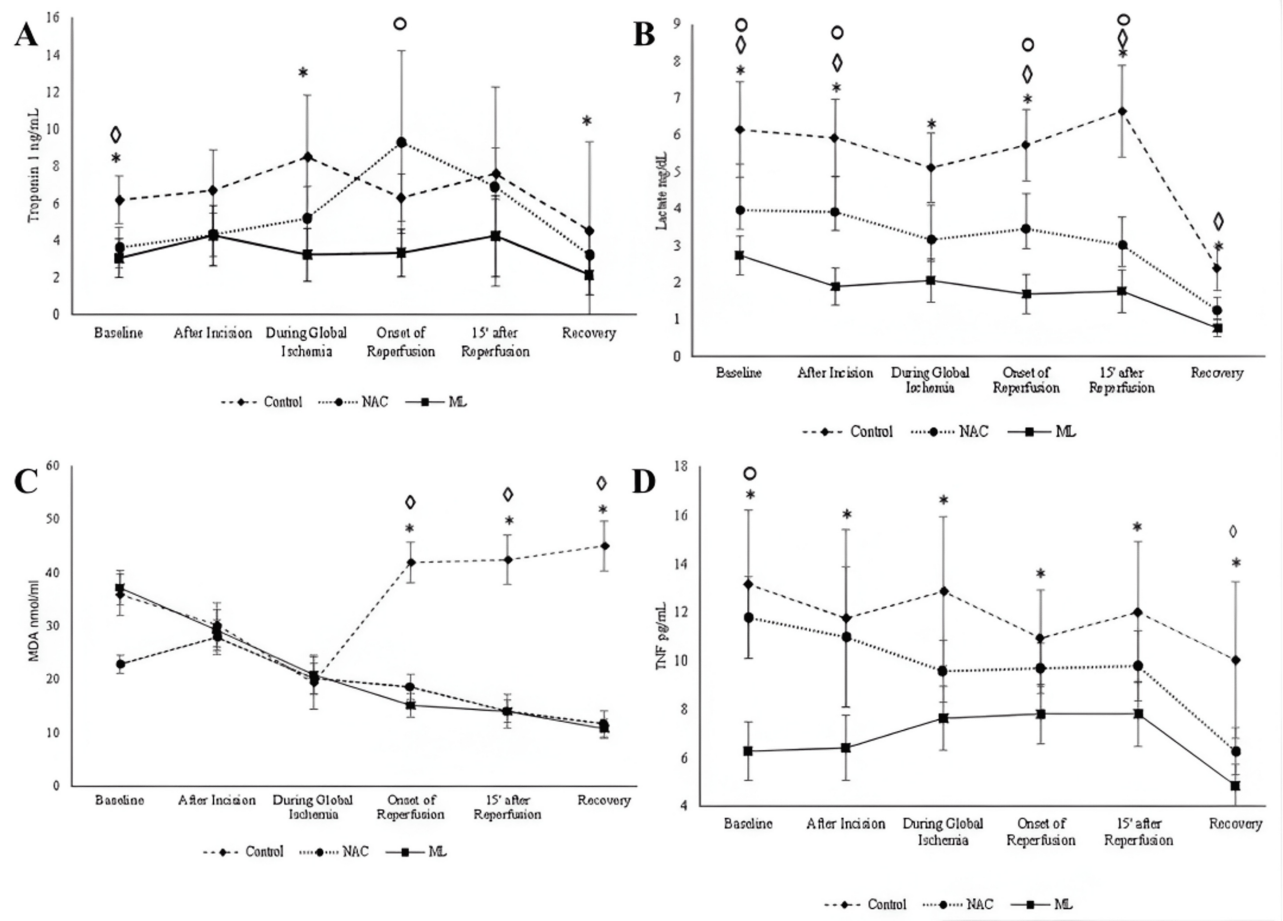
Effect of melatonin and N-acetyl cysteine on postoperative cardiac injury markers, oxidative stress and inflammation. Effect of melatonin and N-acetyl cysteine on (A) and (B) cardiac injury markers, (C) oxidative stress, and (D) inflammation. Means and 95% confidence intervals of four outcome measures over the study period from baseline (before surgery) to recovery stage, following signs on the top of each time-point are indicators of significant P values (P<.05) of pairwise comparisons, control versus ML=∗; control versus NAC=◊; ML versus NAC=○; NAC=N-acetyl cysteine; ML=melatonin. Image Credit: Shafiei et al., 2023 [19]; published under CC BY-ND 4.0, Attribution-NoDerivatives 4.0 International Deed (https://creativecommons.org/licenses/by-nd/4.0/)

In addition to observational findings, interventional studies provide further evidence linking aminothiol metabolism to perioperative redox dynamics. A randomized trial of 88 CABG patients compared supplementation with melatonin, N-acetylcysteine (NAC), or placebo. Both antioxidant therapies reduced oxidative stress and reperfusion injury markers such as malondialdehyde, lactate, troponin I, and tumor necrosis factor-α when measured at ischemia, reperfusion, and postoperative recovery. The ability of NAC to replenish GSH pools highlights the direct relationship between thiol homeostasis and perioperative oxidative stress burden [19]. These perioperative fluctuations in oxidative stress markers across intervention groups are illustrated in Figure 3 [19].

Evidence relating aminothiol measures to clinical outcomes after CABG is growing but remains incomplete. As noted above, a study by Panicker et al. linked preoperative hyperhomocysteinemia to immediate postoperative adverse events [21], while a study by Shammas et al. associated higher homocysteine with cardiovascular death, myocardial infarction, and graft disease at two years [20]. Earlier mechanistic work also ties aminothiol shifts to graft health. A study by Iwama et al. reported that higher plasma homocysteine was associated with saphenous vein graft disease after CABG, suggesting a link between thiol-mediated redox stress and conduit atherosclerosis [40]. Collectively, these data suggest that aminothiols, particularly homocysteine and GSH redox indices, may stratify risk for perioperative complications and longer-term outcomes, although standardized sampling times and multicenter validation are still required.

Recent evidence has extended the relevance of homocysteine beyond plasma measurements into the pericardial compartment. In patients undergoing CABG, homocysteine concentrations were found to be similar in plasma and pericardial fluid, with a strong positive correlation between the two. This distribution likely reflects free diffusion of the small thiol across the epicardium, facilitated by ischemia-induced changes in permeability. Importantly, higher homocysteine levels correlated with markers of adverse cardiac remodeling, including increased left ventricular diameters, atrial size, and left ventricular mass, as well as reduced ejection fraction, underscoring its potential link to impaired contractile function [23].

Clinical implications

The measurement of aminothiols has potential value for risk stratification in patients undergoing CABG. Elevated preoperative plasma homocysteine has been linked to cardiovascular death, nonfatal myocardial infarction, and symptomatic graft disease at two years, indicating that aminothiol profiling may help identify high-risk patients before surgery [20]. Hyperhomocysteinemia has also been associated with acute postoperative complications, reinforcing the role of aminothiols in perioperative prognostication [21]. The clinical implications of these findings are notable. Routine assessment of fasting homocysteine before CABG could help identify high-risk patients who may require intensified perioperative monitoring and risk factor modification. Importantly, the prognostic value of homocysteine was independent of other determinants such as folate and vitamin B12 status, renal function, and systemic inflammation, underscoring its utility as a standalone biomarker. Although trials of homocysteine-lowering with B vitamins have not consistently improved outcomes in general cardiovascular populations, these results suggest that targeted strategies in CABG patients with markedly elevated homocysteine warrant further exploration [22].

**Figure 4:**
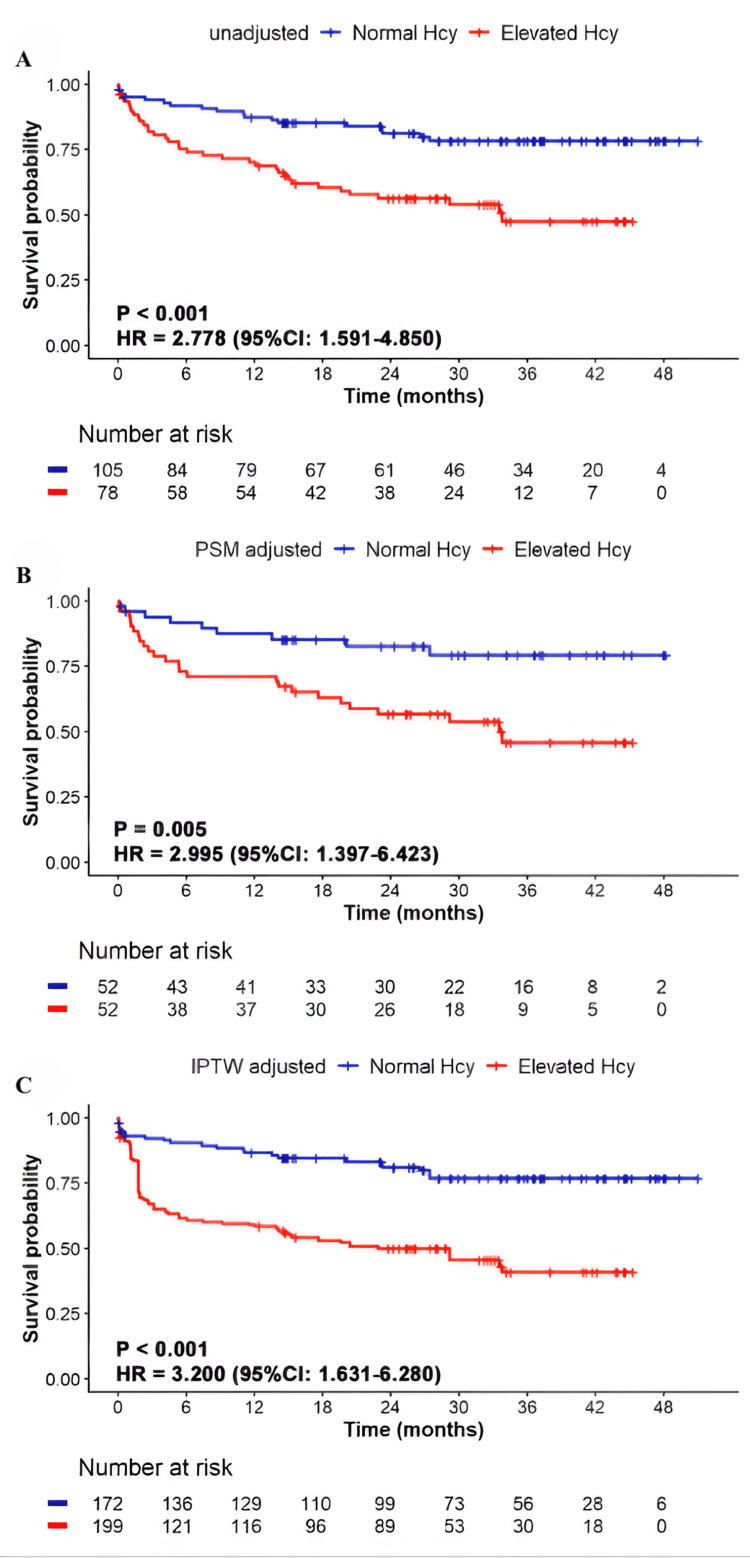
Survival analysis for major adverse cardiovascular events according to Hcy levels Kaplan-Meier survival analysis for MACE according to Hcy levels: (A) unadjusted analysis, (B) PSM adjusted, (C) IPTW adjusted. The survival analysis is performed with the use of the Cox proportional-hazard model. It shows that the elevated Hcy group has a lower survival probability compared to the normal Hcy group, with unadjusted, PSMadjusted, and IPTW-adjusted analyses all confirming an increased risk of MACE associated with higher Hcy levels and demonstrating the robustness of this association after adjusting for confounding factors. Hcy: homocysteine; HR: hazard ratio; CI: confidence interval; PSM: propensity score matching; IPTW: inverse probability of treatment weighting; MACE: major adverse cardiac events. Image Credit: Xiong et al., 2025 [26]; published under CC BY 4.0, Attribution 4.0 International Deed (https://creativecommons.org/licenses/by/4.0/)

The clinical significance of these findings lies in the observation that elevated homocysteine, even within the upper limit of the normal range, was associated with hypertrophic remodeling and functional decline in CABG patients. Moreover, the differential distribution of biomarkers between plasma and pericardial fluid suggests that the latter may provide an underutilized window into perioperative myocardial health. Parallel evidence also implicates other aminothiols in cardiovascular outcomes: high plasma cysteine and cystine levels have been linked with increased cardiovascular disease risk and mortality, whereas reduced GSH indices, including blood GSH and the GSH-to-hemoglobin ratio, were consistently lower in CAD and heart failure patients. These associations reinforce the value of aminothiol profiling as a means of refining perioperative risk assessment and potentially guiding tailored management strategies in CABG [26]. The prognostic value of homocysteine is further illustrated in Figure 4, which shows Kaplan-Meier survival analyses for major adverse cardiovascular events (MACE) in CABG patients stratified by homocysteine levels. Elevated homocysteine was associated with significantly higher rates of adverse outcomes across unadjusted, propensity score-matched, and inverse probability treatment-weighted models [26].

These findings suggest that thiol assessment could complement conventional risk scores to refine patient stratification for both early and late outcomes. The clinical significance of these findings lies in the observation that elevated homocysteine, even within the upper limit of the normal range, was associated with hypertrophic remodeling and functional decline in CABG patients. Moreover, troponin-I concentrations were markedly higher in pericardial fluid compared to plasma, highlighting the pericardial compartment as a sensitive reservoir of myocardial injury biomarkers. These data support the monitoring of plasma homocysteine as part of risk modification strategies in surgical patients, while also pointing to the potential utility of pericardial fluid analyses for detecting subclinical myocardial stress [23].

Aminothiols may also serve as biomarkers for monitoring the efficacy of antioxidant therapies. Evidence from interventional studies further underscores this role. In the trial by Shafiei et al., perioperative administration of NAC and melatonin not only improved biochemical markers of oxidative stress but was also associated with shorter intensive care unit and hospital stays compared to placebo [19]. Although their study was underpowered to assess major clinical outcomes, the results suggest that aminothiol-directed therapies could enhance perioperative recovery while providing a biochemical readout of therapeutic efficacy. A study by El-Hamamsy et al. tested intravenous NAC in CABG patients but did not find significant improvements in clinical outcomes or biochemical markers compared to placebo, illustrating the complexity of translating thiol modulation into measurable benefit [41]. By contrast, vitamin C supplementation has shown more consistent results. Hill et al. demonstrated in a randomized trial that intravenous vitamin C reduced oxidative stress markers during cardiac surgery, and the observed shifts in thiol-disulfide balance supported its protective effect [24]. Rodrigo et al. further emphasized that antioxidant therapies such as NAC, vitamin C, and GSH supplementation act in part by replenishing thiol pools, highlighting aminothiol monitoring as a practical way to track redox-directed interventions [3].

**Figure 5:**
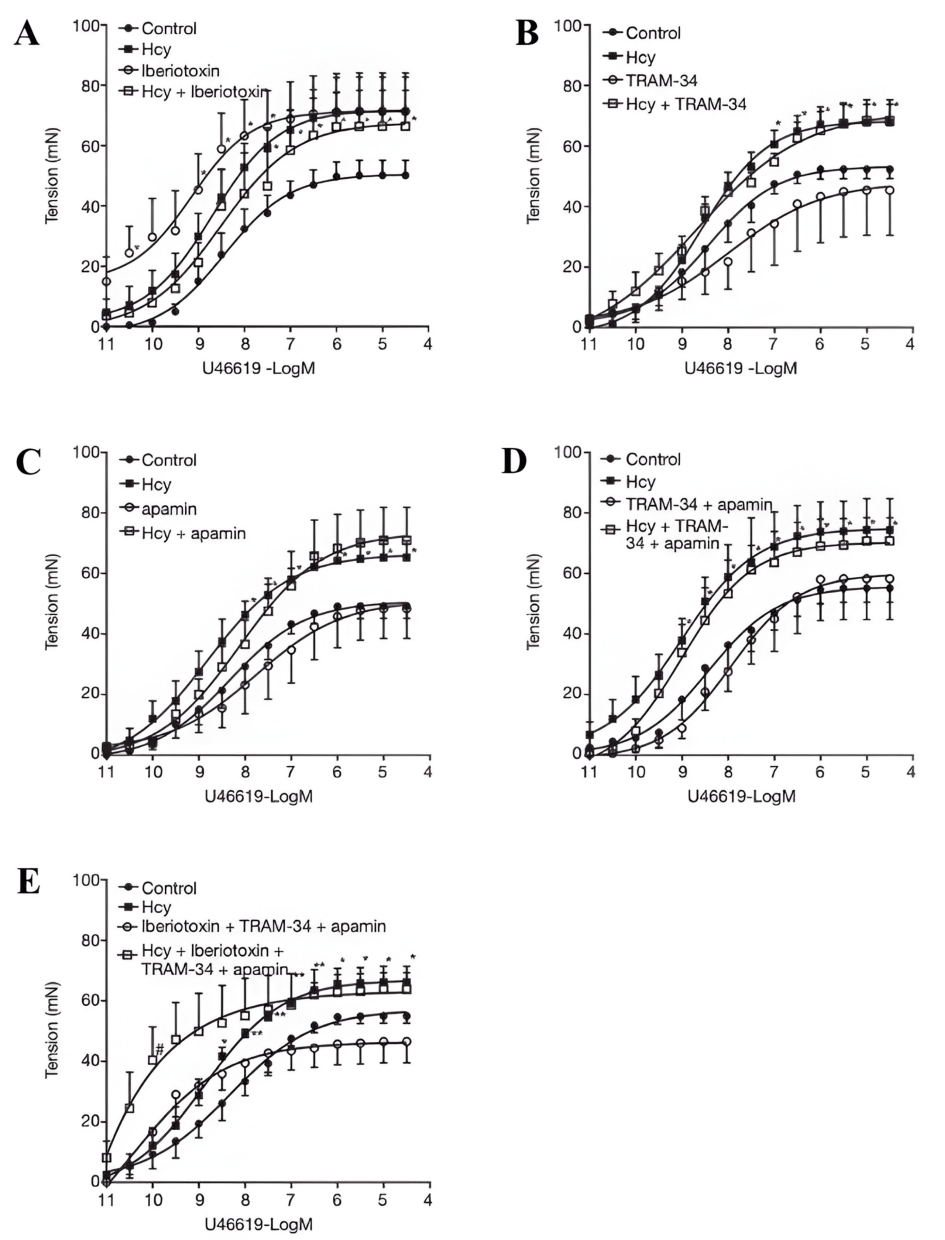
Effect of homocysteine (Hcy) on the role of KCa subtypes in the regulation of internal mammary artery (IMA) contractility Hcy enhances the contractile response of IMA to U46619 and compromises the activity of the BKCa channel subtype in opposing vasoconstriction (A). Neither IKCa nor SKCa channel subtype is involved in U46619-induced contraction in IMAs exposed or not exposed to homocysteine (B,C,D). The KCa channel family is barely involved in the regulation of IMA contractility in hyperhomocysteinemic condition (E). n=6. *P<0.05 vs. Hcy. Iberiotoxin: BKCa channel blocker; TRAM-34: IKCa channel blocker; apamin: SKCa channel blocker Image Source: Sun et al., 2021 [25]; published under CC BY-NC-ND 4.0, Attribution-NonCommercial-NoDerivatives 4.0 International Deed (https://creativecommons.org/licenses/by-nc-nd/4.0/)

Beyond prognosis and therapy monitoring, aminothiols may also inform perioperative management. According to Ivanov et al., changes in GSH redox status after CABG were associated with perioperative glucose levels, suggesting that metabolic control influences oxidative balance [2]. Beyond systemic biomarkers, homocysteine may also impair graft performance at the vascular level. Experimental work on internal mammary artery (IMA) segments obtained from CABG patients demonstrated that homocysteine suppresses BKCa channel-mediated vasodilation by downregulating the β1-subunit, while enhancing thromboxane A2-induced vasoconstriction. Under these conditions, intermediate- and small-conductance KCa channels contribute more strongly to relaxation, but less effectively. This suggests that hyperhomocysteinemia could predispose IMA grafts to vasospasm and impaired flow, with potential implications for graft patency in CABG patients [25]. These effects are illustrated in Figure 5, which shows the attenuation of BKCa channel-mediated relaxation and the enhancement of thromboxane A2-induced constriction in internal mammary artery segments exposed to homocysteine [25].

Oxidative stress has been identified as a driver of inflammation and arrhythmogenesis in cardiac surgery, which implies that aminothiol monitoring could help identify patients at higher risk for POAF or graft dysfunction [14]. In clinical practice, serial measurement of aminothiols could guide targeted antioxidant supplementation, stricter metabolic regulation, or enhanced anti-inflammatory strategies in patients with adverse redox profiles.

Limitations of current evidence

Although aminothiols are promising biomarkers in the context of CABG, current evidence is constrained by several key limitations. Most studies have enrolled relatively small sample sizes, which limits statistical power and the ability to perform meaningful subgroup analyses. For instance, many perioperative investigations into thiol-disulfide dynamics have included fewer than 50 patients [42,43]. As noted in the context of biomarker research, small datasets can lead to wide confidence intervals and unstable associations [42]. A recent report on thiol-disulfide homeostasis similarly highlighted that single-center, underpowered studies limit generalizability and reproducibility of findings [43].

Second, there is substantial heterogeneity in assay methodology. Some investigators have employed high-performance liquid chromatography, others liquid chromatography-mass spectrometry, while others use spectrophotometric thiol assays. Each approach yields different sensitivities and abilities to distinguish reduced from oxidized species. Frijhoff et al. emphasized that reliable measurement of GSSG requires sensitive and validated methods due to its low plasma concentrations [44]. This variability complicates cross-study comparisons.

Third, there is no consensus on reference values for surgical patients. Most thresholds are extrapolated from cardiovascular cohorts outside of the surgical setting, yet CABG involves unique physiological perturbations such as ischemia-reperfusion injury, CPB, and systemic inflammation. For example, Patel et al. showed that thiol redox imbalance predicted mortality in CAD patients, but it remains unclear how such cutoffs translate into perioperative cardiac surgery populations [6]. Similarly, Ertürk et al. demonstrated the utility of dynamic thiol-disulfide homeostasis as an oxidative stress indicator, but without standardized perioperative ranges, interpretation in CABG remains speculative [45].

Fourth, preanalytical variability further limits reliability. Timing of sample collection (preoperative, intraoperative, or postoperative), storage conditions, and stabilization protocols influence measured thiol concentrations. In studies where preoperative interventions were tested, such as short-term sulfur amino acid restriction, changes in thiol status were observed but were highly sensitive to timing and patient characteristics [46]. There is a pressing need for large, multicenter prospective studies. Current evidence is fragmented, with limited ability to establish causal relationships between aminothiol shifts and outcomes such as atrial fibrillation, graft patency, or long-term survival. Future research should prioritize standardized assays, larger cohorts, and harmonized endpoints to validate the prognostic and therapeutic roles of aminothiols in CABG.

Future directions

The next phase of research on aminothiols in CABG should move beyond descriptive associations and address how these biomarkers can be integrated into predictive, mechanistic, and therapeutic frameworks. One promising avenue is the integration of aminothiol profiling into multi-biomarker panels. Current cardiac surgery risk models rely mainly on clinical variables, yet combining aminothiols with markers of inflammation, endothelial dysfunction, and myocardial injury could improve the prediction of postoperative complications. Patel et al. demonstrated that thiol redox imbalance was independently associated with mortality in CAD patients, suggesting that aminothiols contribute incremental prognostic information beyond standard clinical factors [6]. In CABG, such panels could help stratify patients for closer monitoring or tailored perioperative interventions.

Advances in metabolomics and redox proteomics also offer new tools for understanding the role of aminothiols in surgical patients. High-resolution metabolomic profiling has identified distinct thiol-related metabolic signatures linked to oxidative stress and cardiovascular outcomes [47]. Similarly, redox proteomics allows mapping of cysteine oxidation states on proteins, which may uncover mechanisms by which thiol imbalances affect myocardial function or vascular integrity during ischemia-reperfusion [48]. Applying these approaches in CABG populations could yield mechanistic insights while identifying novel therapeutic targets.

Another important question is whether aminothiols are causal mediators of outcomes or merely reflective markers of oxidative stress. Elevated homocysteine, for instance, has been strongly associated with vascular disease, yet randomized trials of homocysteine-lowering therapies have not consistently reduced cardiovascular events [49]. Determining whether perioperative thiol changes actively drive complications such as atrial fibrillation or graft dysfunction, or simply mirror underlying oxidative stress, will require carefully designed mechanistic and interventional studies.

Finally, there is interest in the therapeutic modulation of aminothiol pathways. Strategies to enhance GSH availability, restore thiol-disulfide balance, or reduce pathological thiol oxidation are being investigated in other clinical contexts and could be adapted for CABG. NAC and vitamin C are established thiol-modulating interventions, but emerging approaches such as cysteine supplementation, thioredoxin mimetics, and redox-active pharmacological agents warrant exploration [44,48]. Ultimately, combining biomarker-guided monitoring with targeted therapies could pave the way toward personalized redox medicine in cardiac surgery.

## Conclusions

Aminothiols are emerging as valuable biomarkers of oxidative stress in patients undergoing CABG. Alterations in homocysteine, cysteine, and GSH pathways have been consistently associated with perioperative oxidative burden, myocardial injury, and adverse cardiovascular outcomes. Elevated preoperative homocysteine predicts complications such as myocardial infarction, atrial fibrillation, and long-term graft dysfunction, while changes in GSH redox balance and thiol-disulfide homeostasis reflect intraoperative and postoperative oxidative dynamics. Mechanistic evidence further suggests that aminothiols directly influence the vascular reactivity of graft conduits, highlighting their potential role in graft patency.

Current limitations include small cohorts, heterogeneous assays, and a lack of standardized cut-off values, underscoring the need for large prospective studies. Nevertheless, integration of aminothiol profiling into multi-biomarker panels and perioperative monitoring strategies could refine risk prediction, guide antioxidant therapies, and optimize patient management. Ultimately, aminothiols offer a clinically relevant window into redox biology with potential to improve outcomes in CABG.
